# Quantitative proteomics reveals unique responses to antimicrobial treatments in clinical *Pseudomonas aeruginosa* isolates

**DOI:** 10.1128/msystems.00491-23

**Published:** 2023-08-25

**Authors:** Mara C. Goodyear, Laura Seidel, Jonathan R. Krieger, Jennifer Geddes-McAlister, Roger C. Levesque, Cezar M. Khursigara

**Affiliations:** 1 Department of Molecular and Cellular Biology, University of Guelph, Guelph, Ontario, Canada; 2 Bioinformatics Solutions, Inc, Waterloo, Ontario, Canada; 3 Institut de biologie integrative et des systems (IBIS), Département de microbiologie-infectiologie et d'immunologie, Université Laval, Laval, Quebec, Canada; Chan Zuckerberg Biohub, Stanford, California, USA

**Keywords:** *Pseudomonas aeruginosa*, Liverpool epidemic strain, label-free quantitative proteomics, antibiotics

## Abstract

Epidemic strains of *Pseudomonas aeruginosa* often have increased levels of antibiotic resistance, and these resistance phenotypes are difficult to predict. We used quantitative proteomics to compare the responses of laboratory strain PAO1 and two isolates of the Liverpool epidemic strain (LES) to four antibiotics and hydrogen peroxide. The majority of proteome changes were unique to either one isolate or treatment, but smaller groups of proteins were differentially abundant in more than one sample. Proteins in these shared adaptive responses represent promising avenues for further investigation to understand the contribution of these proteins to resistance and their potential as targets for novel treatments. We observed the largest overlap between the proteome profiles of PAO1 challenged with tobramycin and hydrogen peroxide, and our data support previous work suggesting a role for heat shock protein IbpA in response to tobramycin. Our proteome profiling uncovered extensive changes in LESB58 in response to carbenicillin, with more than 1,000 proteins significantly changed in abundance. This included unique changes in the abundance of proteins involved in cell wall synthesis and division. We used phase contrast microscopy to check for corresponding changes in cell morphology and show that LESB58 maintained shorter cell lengths under treatment with β-lactams. We propose that the ability to maintain the processes of cell wall synthesis and division through changes in protein abundances contributes to the high levels of β-lactam resistance in LESB58.

**IMPORTANCE:**

*Pseudomonas aeruginosa* is an important pathogen often associated with hospital-acquired infections and chronic lung infections in people with cystic fibrosis. *P. aeruginosa* possesses a wide array of intrinsic and adaptive mechanisms of antibiotic resistance, and the regulation of these mechanisms is complex. Label-free quantitative proteomics is a powerful tool to compare susceptible and resistant strains of bacteria and their responses to antibiotic treatments. Here we compare the proteomes of three isolates of *P. aeruginosa* with different antibiotic resistance profiles in response to five challenge conditions. We uncover unique and shared proteome changes for the widely used laboratory strain PAO1 and two isolates of the Liverpool epidemic strain of *P. aeruginosa*, LESlike1 and LESB58. Our data set provides insight into antibiotic resistance in clinically relevant *Pseudomonas* isolates and highlights proteins, including those with uncharacterized functions, which can be further investigated for their role in adaptive responses to antibiotic treatments.

## INTRODUCTION

*Pseudomonas aeruginosa* is one of the most common causes of hospital-acquired infections, often affecting people with burn wounds and those using mechanical ventilators or catheters (1). *P. aeruginosa* also causes chronic infections in the lungs of people with cystic fibrosis (pwCF). While these lung infections are caused by various bacteria and fungi, they are often dominated by *P. aeruginosa*. Alarmingly, *P. aeruginosa* isolates from pwCF have been found to be resistant to almost all classes of antibiotics used in CF treatments (2). The genome of *P. aeruginosa* encodes a range of antibiotic resistance mechanisms and regulatory systems that allow it to survive treatment in the clinic and adapt to infection conditions. Understanding the mechanisms that allow *P. aeruginosa* to survive antibiotic treatments is essential to our ability to develop novel therapies and better predict which antibiotics will be effective in eradicating infections in the future.

Proteomics provides a global understanding of protein dynamics involved in bacterial responses to growth conditions. In *P. aeruginosa*, proteomics has been used to study the differences between cells growing planktonically or in biofilms to successfully identify the proteins important in each mode of growth, in the stages of biofilm formation, and that may contribute to the increased antibiotic resistance of biofilms (3 – 7). To further our understanding of antibiotic resistance mechanisms, proteomics can be used to compare susceptible and resistant strains and to characterize the effects of antibiotic treatment on the proteome (8). Previous proteomic studies have characterized the response of *P. aeruginosa* to antibiotics or stress conditions using label-free quantitative proteomics [tobramycin (9), hypoxic stress (10), and copper (11)] or two-dimensional (2D) gel-based proteomics [ciprofloxacin (12), azithromycin (13), oxidative stress (14), and a panel of 12 antibiotics (15)]. By characterizing the proteome profiles in response to these different antibiotics and stress conditions, these studies have identified proteins that may contribute to the antibiotic resistance of *P. aeruginosa* or its survival in lung infections and have highlighted how antibiotic treatments impact other essential functions, such as virulence. These studies have also described potential functions for previously uncharacterized proteins and identified marker proteins for the proteome response to different antibiotic classes. Most of these proteomics studies have focused on the commonly used laboratory strain PAO1 or gene mutants in a PAO1 background, while only one study included two clinical isolates of *P. aeruginosa* (10). Performing such analyses on clinical isolates is critical to understand the complexity of the biological system and to identify specific proteins that may confer resistance and/or represent new treatment targets.

We previously characterized clinical isolates of the Liverpool epidemic strain (LES) of *P. aeruginosa* and showed they had increased resistance to most classes of antibiotics compared to laboratory strains and that a subset of LES isolates has increased resistance to β-lactam antibiotics (16). The LES was the first reported transmissible strain of *P. aeruginosa* and has been associated with worse health outcomes (17, 18). We also recently used label-free quantitative proteomics to compare β-lactam-susceptible (PAO1 and LESlike1) and -resistant (LESB58) isolates in the absence of antibiotic treatment (19). This information provides critical new insight into differences in the abundance of proteins and pathways involved in antibiotic resistance in these isolates, which is important for complementing genomic data to understand and predict resistance phenotypes.

In the present study, we profile changes to proteome signatures when the same laboratory and clinical isolates are exposed to antibiotics. To determine treatment conditions, we developed a workflow that first used susceptibility testing and time-kill assays. We used these data to select the concentrations and lengths of treatment to challenge PAO1, LESlike1, and LESB58 with three β-lactams (aztreonam, carbenicillin, and piperacillin), the aminoglycoside tobramycin, and H_2_O_2_ (stress response control). Here, we describe proteome remodeling caused by non-β-lactam versus β-lactam treatments and highlight commonalities and distinctions between treatments and isolates. We uncover novel findings of antibiotic effects on proteome signatures among the strains, and we report specific proteins and pathways that serve as benchmarks for antibiotic resistance, warranting further exploration.

## RESULTS

### Time-kill assays determine optimal proteome profiling conditions

**Fig 1:**
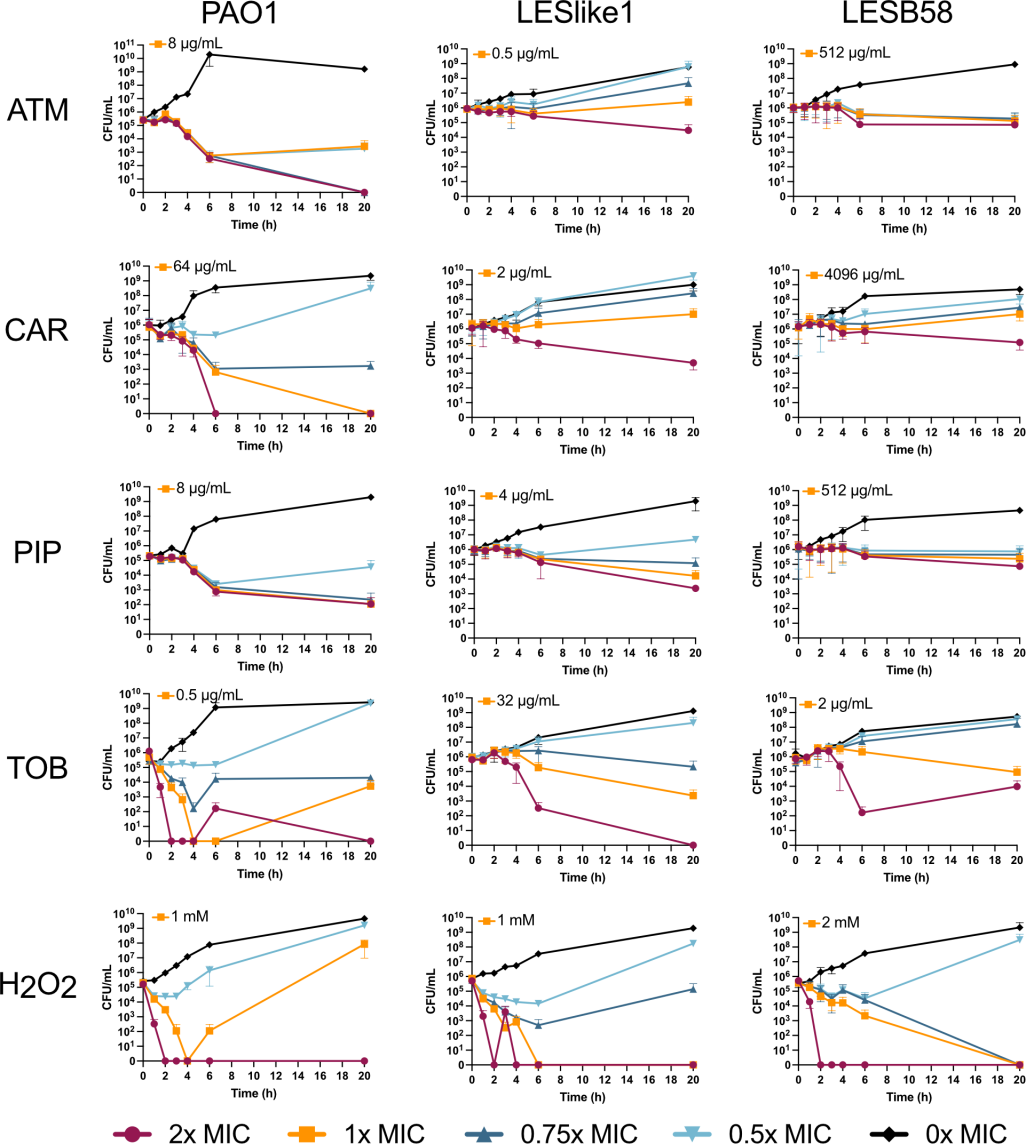
Time-kill curves for PAO1, LESlike1, and LESB58. CFU/mL values over time are shown for untreated samples and cultures exposed to H_2_O_2_, tobramycin (TOB), aztreonam (ATM), carbenicillin (CAR), or piperacillin (PIP) at 2×, 1×, 0.75×, and 0.5× their MIC values. Values are reported as the mean ± SD for biological replicates (*n* = 3 biological replicates for PAO1 and *n* = 2 biological replicates for LESlike1 and LESB58, three technical replicates per biological replicate). The MIC value for each isolate and treatment is indicated in the top left corner of each graph. For the proteomics experiments, isolates were treated at their MIC values (orange line) for 2 hours (H_2_O_2_ and TOB) or 4 hours (ATM, CAR, PIP).

Our previous work characterizing the antibiotic susceptibility of LES isolates showed that a subset of isolates had increased resistance to β-lactam antibiotics (16). Here, we aimed to further investigate one β-lactam-resistant and one β-lactam-susceptible LES isolate by comparing the proteome profiles of LES isolates with each other and with PAO1, under both untreated and antibiotic challenge conditions. LESB58 was selected as an isolate that showed increased resistance to multiple classes of β-lactams (penicillins, cephalosporins, penems, and monobactams), and LESlike1 was selected as an isolate that did not show increased resistance to β-lactam antibiotics. For challenge conditions, three β-lactam antibiotics were selected (aztreonam, carbenicillin, and piperacillin) and we also chose to challenge the isolates with tobramycin as a non-β-lactam condition and with H_2_O_2_ to generate a general stress response. We used time-kill assays to quantify the effect of different antibiotic concentrations on cell density over time and to inform optimal conditions for subsequent proteome profiling. Initial proteome profiling of cultures exposed to sub-MICs of antibiotics for 1 hour showed very few changes in the proteome (data not shown). Therefore, we used the time-kill assays to identify later time points and higher concentrations of each treatment that could be applied to detect greater proteome changes while maintaining enough cells for proteomics sample preparation. Time-kill assay curves are shown in Fig. 1. Based on the time-kill assays for PAO1, we chose to treat samples at the MIC for 2 or 4 hours and grew untreated controls for both time points. In PAO1, we saw that H_2_O_2_ and tobramycin killed the cultures faster than the β-lactam treatments and resulted in no detectable CFUs after 4 hours at 1× MIC. We therefore chose 2-hour exposures for H_2_O_2_ and tobramycin. For the β-lactam treatments, we chose a 4-hour exposure given an observed decrease in growth of PAO1, with a remaining cell density of ~10^4^ CFU/mL. In the LES isolates, the β-lactam treatments had less impact on CFU/mL values during the first 6 hours compared to PAO1. Even at 2× and 1× the MIC of the β-lactam treatments, LESlike1 and LESB58 maintained 10^5^–10^6^ CFU/mL after 6 hours, while PAO1 CFU/mL values for the β-lactam treatments were closer to 10^3^ (Fig. 1).

### Proteome profiling of *P. aeruginosa* isolates exposed to antibiotics

**Fig 2:**
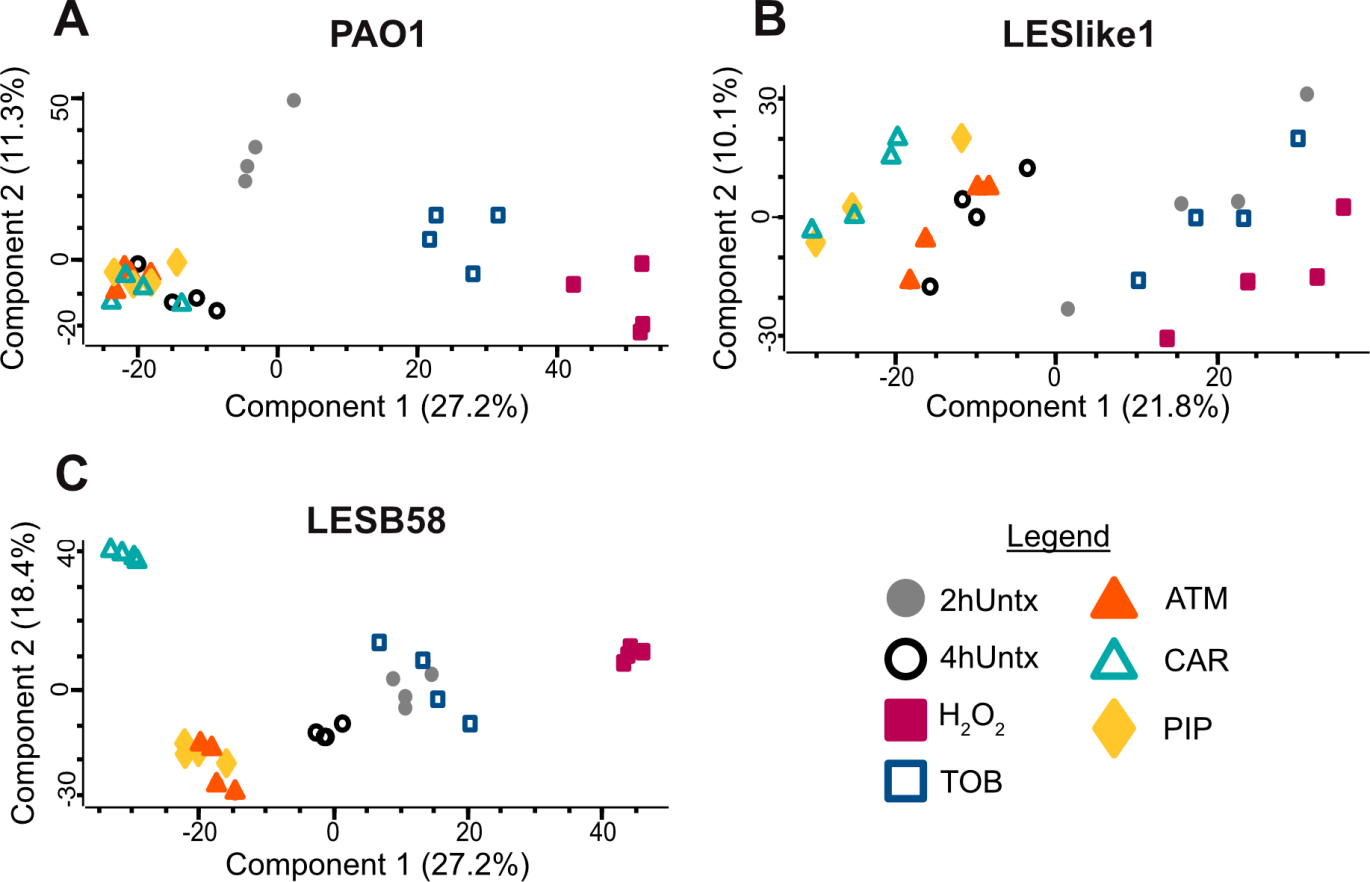
Principal component analysis (PCA). PCA at the experiment level for (**A**) PAO1, (**B**) LESlike1, and (**C**) LESB58 samples. Symbols represent untreated controls collected at 2 hours (2hUntx, solid gray circles) and 4 hours (4hUntx, black circles) and samples treated with H_2_O_2_ (solid maroon squares), tobramycin (TOB, dark blue squares), aztreonam (ATM, solid orange triangles), carbenicillin (CAR, aqua triangles), and piperacillin (PIP, yellow diamonds).

Using label-free quantitative proteomics, we identified 3,019 proteins across all samples with replicate reproducibility for groups of quadruplicate biological replicates within each isolate >93% (Fig. S1). Proteome profiles for each treated isolate were compared with their time-matched untreated control (2 or 4 hours). After filtering for valid values (i.e., protein must be identified in three out of four biological replicates), missing values were imputed from a normal distribution and the number of proteins remaining for statistical analysis in each isolate was 2,631 in PAO1, 2,634 in LESlike1, and 2,854 in LESB58. Coefficients of variation were <0.05 for more than 81% of proteins in each of the 21 groups of biological replicates (Table S1; Fig. S2). A principal component analysis (PCA) for each isolate showed separation between 2- and 4-hour samples, as well as treatment-driven clustering (Fig. 2). In PAO1, the 4-hour samples clustered together (including the untreated samples) and were separated from the H_2_O_2_ and tobramycin samples (Fig. 2A). In LESlike1, the samples did not cluster as tightly as in PAO1 and LESB58 and only showed separation between the time points (Fig. 2B). In LESB58, there was an additional separation between the carbenicillin-treated samples and the other β-lactam treatments (Fig. 2C). PCA plots generated separately for the 2- or 4-hour samples for each isolate show similar patterns of clustering for the conditions at each time point (Fig. S3).

**Fig 3:**
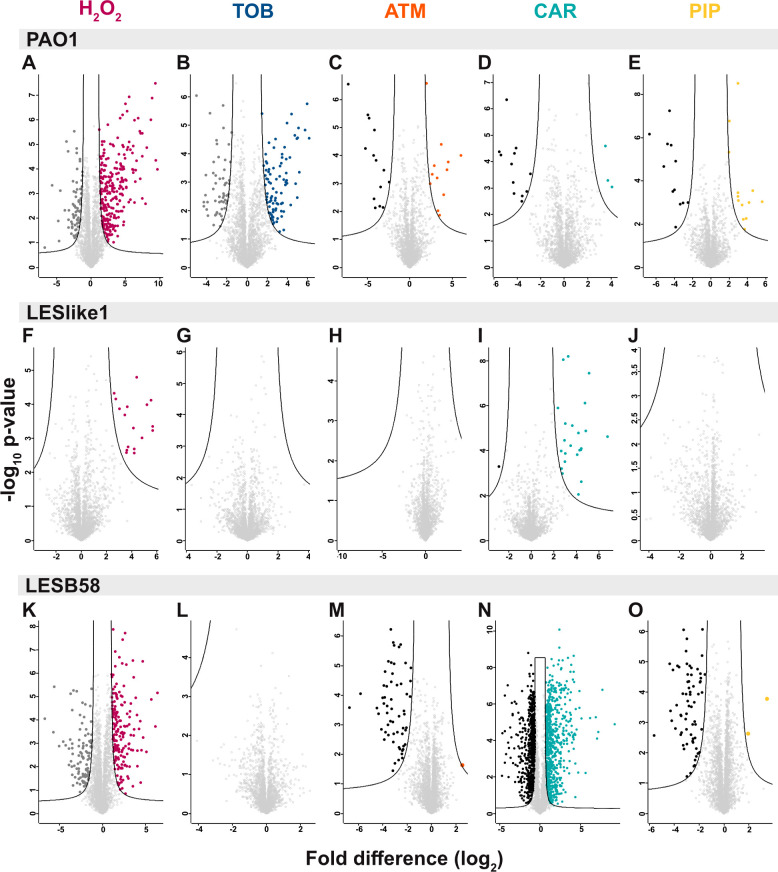
Proteome profiling of treated cultures. Volcano plots show the results of Student’s *t*-tests (*P* ≤ 0.05, FDR = 0.05, S0 = 1) for the comparison of treated samples with their time-matched untreated control for (**A–E**) AO1, (**F–J**) ESlike1, and (**K–O**) ESB58. Proteins that are significantly decreased in abundance in the treatment are represented by points outside the cutoff curve on the left (2 hours, dark gray; 4 hours, black), while proteins significantly increased in abundance in the treatments are represented by the colored points outside the cutoff curve on the right (H_2_O_2_, maroon; TOB, tobramycin, dark blue; ATM, aztreonam, orange; CAR, carbenicillin, aqua; and PIP, piperacillin, yellow). Note that *y*-axis and *x*-axis values show the most appropriate range for each plot and are not equivalent in each of A–O.

To identify proteins with significantly different abundances on exposure to a treatment, we used Student’s *t*-tests [*P* ≤ 0.05, false discovery rate (FDR) = 0.05, S0 = 1] (Table S2 to S4). Volcano plots (Fig. 3A through E) show that PAO1 was the only isolate to have proteins with significantly different abundances in all treatments compared to their time-matched untreated controls. LESlike1 showed the fewest changes and only had proteins with significantly different abundances in H_2_O_2_ and carbenicillin (Fig. 3F through J), while LESB58 had proteins significantly altered in abundance in all treatments except for tobramycin (Fig. 3K through O). The *t*-test results for LESB58 treated with carbenicillin stood out the most with the highest number of proteins with significantly different abundances compared to the untreated control (644 proteins increased in abundance and 590 decreased in abundance). In separate analyses, we have compared the abundances of proteins across the untreated samples for the three isolates at 2 and 4 hours. The results of the isolated comparison (4-hour samples) have been previously published (19), and the lists of proteins that were significantly changed in abundance in the isolated comparisons at 2 or 4 hours are available in Table S5. We also completed a cluster analysis in Perseus based on protein intensities (without imputation) for all treated and untreated samples. This analysis highlighted clusters with different patterns of protein intensities and detection between the three isolates. Large clusters of proteins identified across all samples mainly contain proteins involved in essential cellular processes, such as transport, transcription, translation, and metabolic pathways, while smaller clusters of proteins with distinct patterns in protein intensities contain proteins with functions, such as adaptation, virulence, and antibiotic resistance (Fig. S4).

**Fig 4:**
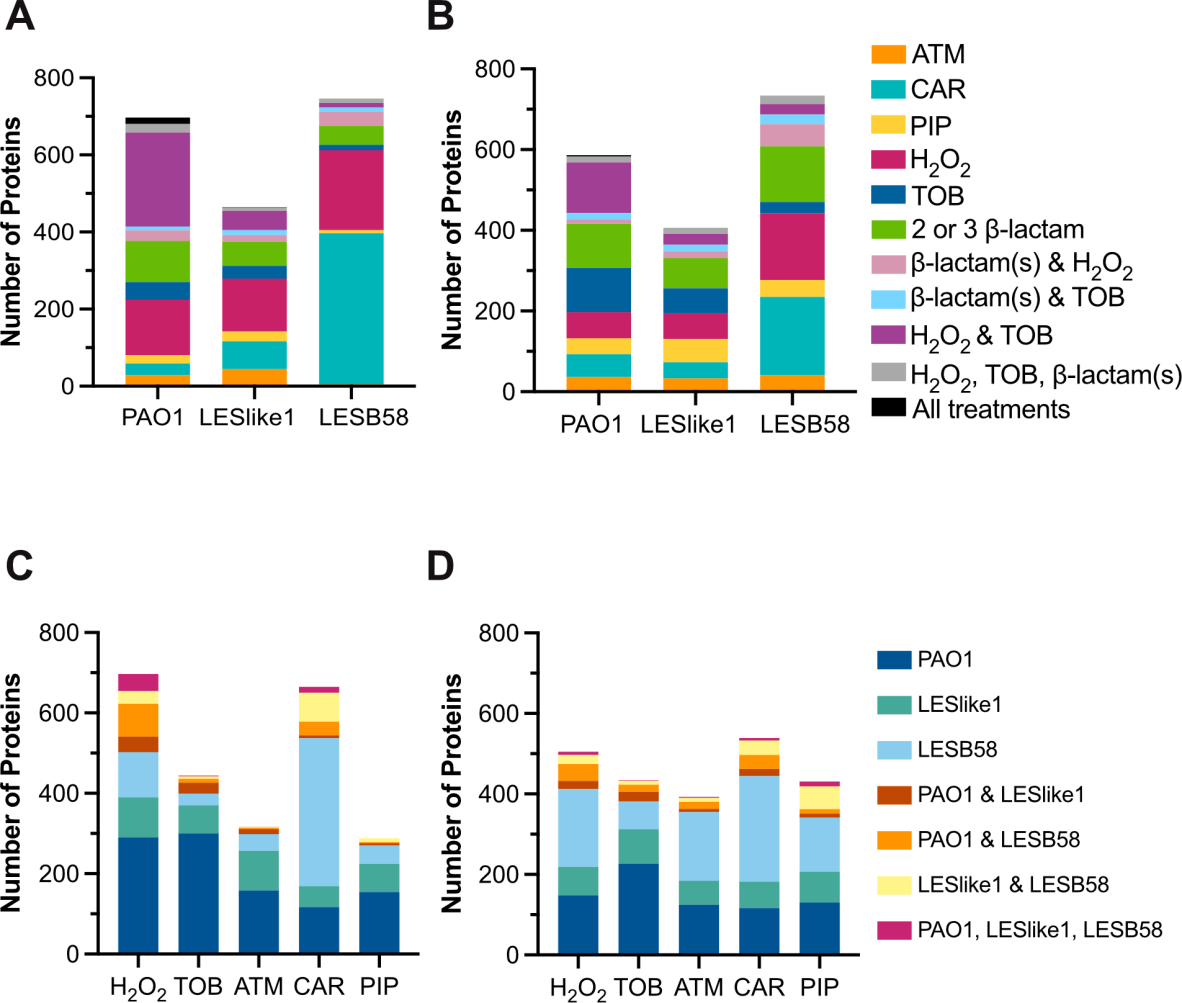
Distribution of proteins with ≥2-fold changes in abundance. Graphs show overlap between treatments for proteins (**A**) increased and (**B**) decreased in abundance ≥2-fold and the number of proteins (**C**) increased and (**D**) decreased in abundance with ≥2-fold changes in one or more isolate. ATM, aztreonam; CAR, carbenicillin; PIP, piperacillin; TOB, tobramycin.

**Fig 5:**
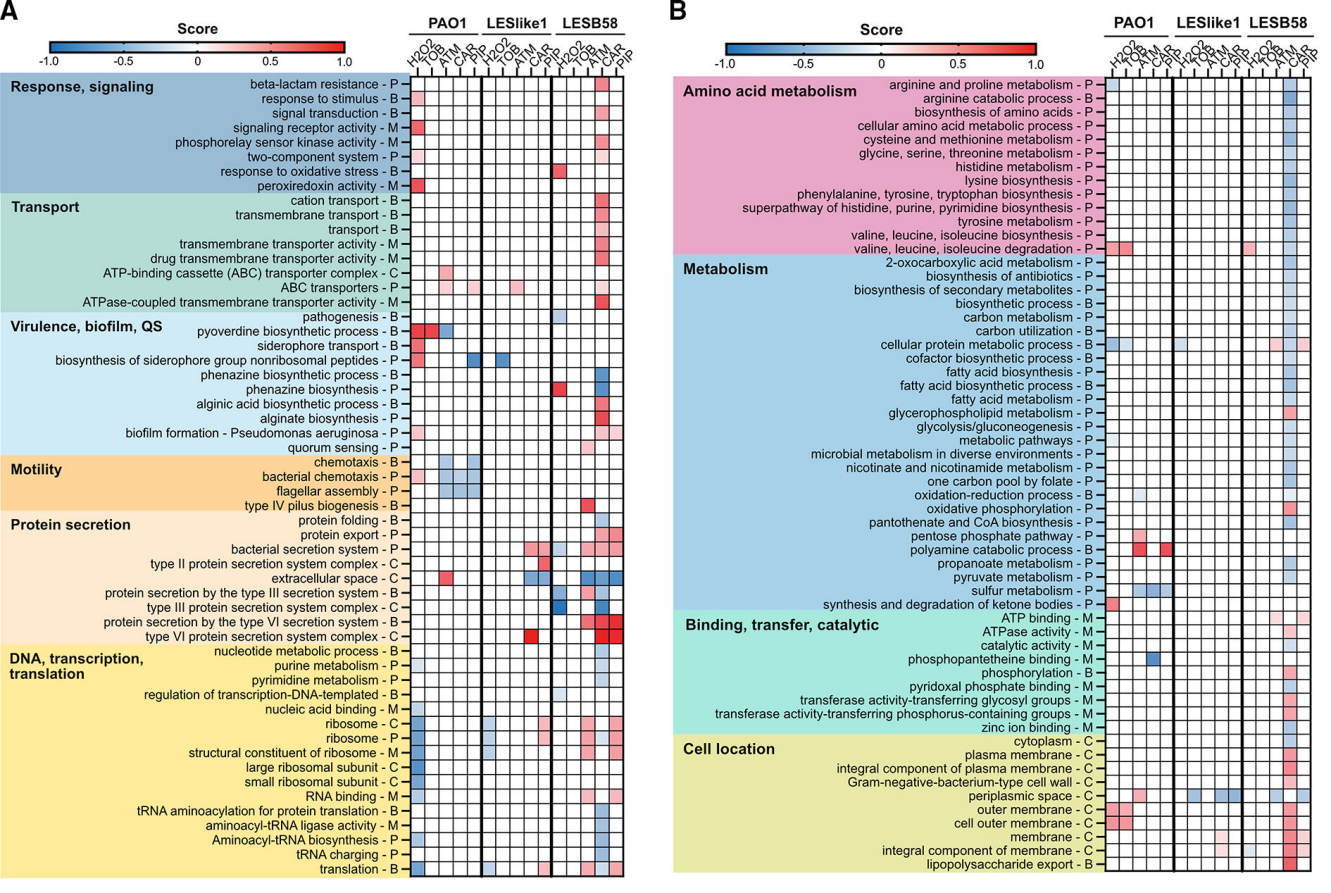
1D annotation enrichment analysis of treated samples. Heat plot shows GO terms (B, biological process; C, cellular component; M, molecular function) and pathway terms (P-KEGG and PseudoCyc) that were enriched for proteins that were increased (positive score, red) or decreased (negative score, blue) in abundance in each comparison. QS, quorum sensing; TOB, tobramycin; ATM, aztreonam; CAR, carbenicillin; PIP, piperacillin.

Bar graphs were used to visualize overlap in proteins with altered abundance profiles (either increased or decreased in abundance) in more than one isolate or treatment (Fig. 4). We included all proteins with ≥2-fold changes in abundance because not all *t*-tests had statistically significant results and created graphs to show overlap among treatments in the same isolate (Fig. 4A and B) and graphs to show overlap between isolates exposed to the same treatment (Fig. 4C and D). This analysis confirmed the trend of fewer proteome changes for LESlike1 compared to the other isolates. In PAO1, a number of proteins were increased or decreased in abundance in both H_2_O_2_ and tobramycin treatments (Fig. 4A and B, purple bar). Proteins changed in abundance in treatments with an antibiotic, and with H_2_O_2_, may be important in stress responses of *P. aeruginosa*, regardless of the cause. In each isolate, there was also a group of proteins affected by two or more of the β-lactam treatments (Fig. 4A and B, green bar). Very few proteins increased or decreased with ≥2-fold changes in all five treatments (Fig. 4A and B, black bar). When looking at the response to the same treatment across PAO1, LESLike1, and LESB58, most proteins were increased or decreased with ≥2-fold changes in only one isolate (Fig. 4C and D). To understand the proteins and pathways that were affected in the different conditions, we next performed a one-dimensional (1D) annotation enrichment analysis (i.e., tests for every annotation term whether the corresponding numerical values have a tendency to be systematically larger or smaller than the global distribution of the values for all proteins) (Fig. 5) and examined proteins with high fold changes in abundance. The 1D annotation enrichment analysis provided a broad overview of the functions impacted by the treatments and highlights the diversity of cellular systems that were affected. In the following sections, we describe the main changes observed in the responses to non-β-lactam treatments and to β-lactam treatments.

### Responses to non-β-lactam treatments

To identify whether proteome changes were specific to challenge with β-lactam antibiotics, we included two non-β-lactam controls. We challenged bacteria with tobramycin, an aminoglycoside which inhibits protein synthesis, and H_2_O_2_, which causes oxidative stress. In addition to acting as non-β-lactam controls, we describe and compare the proteome changes in PAO1, LESlike1, and LESB58 caused by these treatments.

### Hydrogen peroxide leads to shared oxidative stress response and unique changes in iron and secretion

As expected, when all three isolates were exposed to H_2_O_2_, there was an increase in the abundance of proteins involved in the response to oxidative stress, as shown previously for PAO1 (14, 20, 21). Terms enriched in the 1D annotation enrichment analysis in H_2_O_2_ treatments included the gene ontology (GO)—biological process (GOBP) term response to stimulus and GO—molecular function (GOMF) term peroxiredoxin activity in PAO1 and the GOBP term response to oxidative stress in LESB58 (Fig. 5). Proteins from these categories that were significantly increased in abundance in all three isolates include catalase KatB, alkyl hydroperoxide reductase AhpB, and alkyl hydroperoxide reductase subunit AhpF. Three other proteins from these categories were significantly increased in abundance in PAO1 and LESB58 and increased >2-fold in LESlike1, the catalase KatA, alkyl hydroperoxide reductase subunit AhpC, and superoxide dismutase (SodM). Two other proteins that were not in these enriched categories but were also significantly increased in abundance in all three isolates exposed to H_2_O_2_ were RecN, which is involved in DNA repair, and PA1466, a putative glutathione *S*-transferase.

In all three isolates, we also saw an increase in phage-related proteins in the H_2_O_2_ condition. In PAO1, among the proteins with the 20 highest fold increases in abundance in the H_2_O_2_ treatment, there were 12 proteins with the *Pseudomonas aeruginosa* Community Annotation Project (PseudoCAP) annotation related to phage, transposon, or plasmid (PA0618, PA0620, PA0622-PA0625, PA0628-PA0629, PA0633-PA0634, PA0638, PA0641). In LESB58, there were three phage-related proteins among the 20 proteins with the highest fold increases in abundance and in LESlike1 five out of the 17 proteins that were significantly increased in abundance were related to phage. In LESlike1, this included two proteins that were also significantly increased in abundance in PAO1, PA0622-PA0623, which correspond to the LESlike1 loci T225_RS03150 and T225_RS03155. The other phage-related proteins in the LES isolates were identified by UPI number (UniParc identification) indicating that they do not have a blast hit when searched against the PAO1 proteome.

In PAO1, another noticeable change on H_2_O_2_ exposure was an increase in the abundance of proteins related to pyoverdine biosynthesis and iron/siderophore transport, which is consistent with other studies (14, 20, 21). There were five terms enriched in PAO1 related to these functions, including the GOBP terms response to stimulus, pyoverdine biosynthetic process, and siderophore transport; the GOMF term signaling receptor activity; and the KEGG term biosynthesis of siderophore group nonribosomal peptides (Fig. 5). In these five categories, there were 27 different proteins significantly increased in abundance in PAO1 exposed to H_2_O_2_, including PvdADEFHJLNOPQ, FpvAB, FptA, FecA, HasR, PirA, OpmQ, PchABDEFG, PA1365, PA2368, and PA2393. In LESB58, there were two proteins among the 20 proteins with the highest fold change increase in abundance that were related to pyoverdine and iron transport (PvdQ and PhuS). However, for the 27 proteins listed above for PAO1, not all were identified in the LES isolates in the H_2_O_2_ condition, and the ones that were identified showed overall lower fold change increases or decreased abundances in the LES on exposure to H_2_O_2_.

In LESB58 exposed to H_2_O_2_, there was a decrease in the abundance of proteins related to the type III secretion system (T3SS). Four annotations related to the T3SS were enriched for proteins decreased in abundance, including the GOBP terms protein secretion by the T3SS and pathogenesis, the GOCC term type III protein secretion system complex, and the KEGG term bacterial secretion system (Fig. 5). There were 16 proteins in these categories related to the T3SS that were significantly decreased in abundance in LESB58 exposed to H_2_O_2_, including ExoSTY, PopBN, PcrHV, PscEFGHP, ExsC, and PA3842.

### PAO1 response to tobramycin is similar to H_2_O_2_ treatment, while LES isolates show little response to tobramycin

In the LES isolates treated with tobramycin, there were no proteins with significantly different abundances compared to the untreated controls (Fig. 3G and L). PAO1 showed a greater response to the tobramycin treatment and consistency with the H_2_O_2_ response. In PAO1 treated with tobramycin, 85 of the 88 proteins significantly increased in abundance were also significantly increased in abundance in the H_2_O_2_ treatment, and 33 of the 57 proteins significantly decreased in abundance were also significantly decreased in abundance in the H_2_O_2_ treatment. As in the response to H_2_O_2_, in PAO1 exposed to tobramycin the GOBP term pyoverdine biosynthetic process was enriched for proteins significantly increased in abundance. Among the 20 proteins with the highest fold change increases in abundance in PAO1 treated with tobramycin were 11 related to pyoverdine and siderophores, including PvdDEHILMNP (Table S2). In contrast, in LESlike1, the KEGG term biosynthesis of siderophore nonribosomal peptides was enriched for proteins decreased in abundance in the tobramycin treatment and some of the proteins with the highest fold change decreases in abundance are related to pyoverdine and iron transport.

### Responses to β-lactam treatments: aztreonam, carbenicillin, and piperacillin

To compare the responses of PAO1 and the LES isolates to β-lactams, we used three β-lactam treatments: aztreonam, carbenicillin, and piperacillin. These antibiotics target penicillin-binding proteins to interfere with peptidoglycan and cell wall synthesis (22). Aztreonam targets penicillin-binding protein 3 (PBP3/FtsI), while carbenicillin and piperacillin target a wider range of PBPs (DrugBank entries: ATM, DB00355; CAR, DB00578; PIP, DB00319) (23). We have previously shown that LESB58 exhibits MIC values that are 60 to 1,000 times higher for the chosen β-lactam antibiotics compared to PAO1 and LESlike1 (16). In PAO1, 15–25 proteins were significantly different in abundance in each of the β-lactam treatments (Fig. 3). There were several terms enriched in more than one β-lactam condition for PAO1 (Fig. 5). For proteins that increased in abundance, the terms ABC transporters and polyamine catabolic process were enriched, and for proteins that decreased in abundance terms related to chemotaxis and sulfur metabolism were enriched. Proteins that were significantly different in abundance in more than one β-lactam treatment in PAO1 are listed in Table 1.

**TABLE 1:** Proteins with significantly different abundances in two or three β-lactam treatments in PAO1^*a*^

Gene locus	Protein name	Function	Fold difference (log_2_) ATM^*b*^	Fold difference (log_2_) CAR^*c*^	Fold difference (log_2_) PIP^*d*^
Increased					
PA0314	PA0314	ABC transporters	**4.51**	**3.74**	**4.55**
PA0315	PA0315	Hypothetical, unknown	**4.37**	2.71	**4.17**
PA0388	PA0388	Hypothetical, unknown	**2.86**	1.49	**2.95**
PA1634	KdpB	Potassium ion transport	**1.97**	2.06	**2.00**
PA3227	PpiA	Cellular protein metabolic process	**4.00**	3.43	**3.84**
PA3787	PA3787	Hypothetical, unknown	**3.30**	2.98	**3.55**
PA4571	PA4571	Electron transfer activity	**2.61**	**3.51**	**2.94**
PA4738	PA4738	Hypothetical, unknown	**3.68**	2.37	**3.38**
PA4739	PA4739	Hypothetical, unknown	**6.04**	**4.09**	**5.60**
PA5313	GabT2	Polyamine catabolic process	**3.20**	2.87	**3.00**
Decreased					
PA0284	PA0284	Hypothetical, unknown	−**4.20**	−**4.11**	−**5.15**
PA0619	PA0619	Related to phage	−**3.09**	−**3.19**	−**2.53**
PA0633	PA0633	Related to phage	−**3.99**	−**3.63**	−**3.36**
PA1116	PA1116	Hypothetical, unknown	−**3.18**	−2.87	−**3.86**
PA2788	PA2788	Signal transduction, probable chemotaxis transducer	−**4.27**	−**5.41**	−**3.86**
PA3441	PA3441	Molybdate ion transport	−3.24	−**3.58**	−**3.03**
PA3444	SsuD	Sulfur metabolism	−**7.29**	−**5.56**	−**6.73**
PA3446	PA3446	Sulfur metabolism	−**5.23**	−**4.51**	−**4.34**
PA3450	LsfA	Cell redox homeostasis	−**4.80**	−**4.92**	−**4.51**
PA4078	PA4078	Catalytic activity, probable nonribosomal peptide synthetase	−**4.09**	−**4.37**	−**4.16**
PA4131	PA4131	Oxidation-reduction process, probable iron-sulfur protein	−**4.96**	−**4.27**	−**4.77**

^*a*^ Fold differences in bold indicate significantly different abundances compared to the untreated control (Student’s *t*-test, *P* < 0.05, FDR = 0.05, S0 = 1).

^*b*^ ATM, aztreonam.

^*c*^ CAR, carbenicillin.

^*d*^ PIP, piperacillin.

For β-lactam treatments in LESlike1, there were only proteins significantly different in abundance in the Student’s *t*-test for carbenicillin. Only one protein, PA2266, which is a probable cytochrome c precursor, was significantly decreased in abundance. Among the other proteins with the 20 highest fold change decreases in abundance in LESlike1 treated with carbenicillin were two proteins, PpiA and PA4739, that showed the opposite trend in PAO1 where they were significantly increased in abundance in multiple β-lactam treatments (Table 1). Unexpectedly, the β-lactamase AmpC was also among the proteins with the 20 highest fold change decreases in abundance in LESlike1 treated with carbenicillin. We previously showed that AmpC abundance is lower in LESlike1 compared to the β-lactam-resistant isolate LESB58 even under untreated conditions (19). In *P. aeruginosa,* AmpC production is often induced on exposure to β-lactams through an AmpR-dependent signaling pathway (24). This may suggest an altered response to β-lactam exposure in LESlike1, which could account for its lower resistance to β-lactams. Of the 23 proteins significantly increased in abundance in LESlike1 treated with carbenicillin, 13 were related to the type six secretion system (T6SS). This increase in the T6SS was seen in both LES isolates and appears to be a common response to all three β-lactam treatments. In both LESlike1 and LESB58, annotations related to the T6SS were enriched in one or more β-lactam treatments (Fig. 5). The proteins with these annotations that were increased in abundance are mainly encoded within two of the three T6SS gene clusters in *P. aeruginosa* (HSI-1 and HSI-2) and form part of the T6SS apparatus structure (25).

### Response of LESB58 to β-lactams

**Fig 6:**
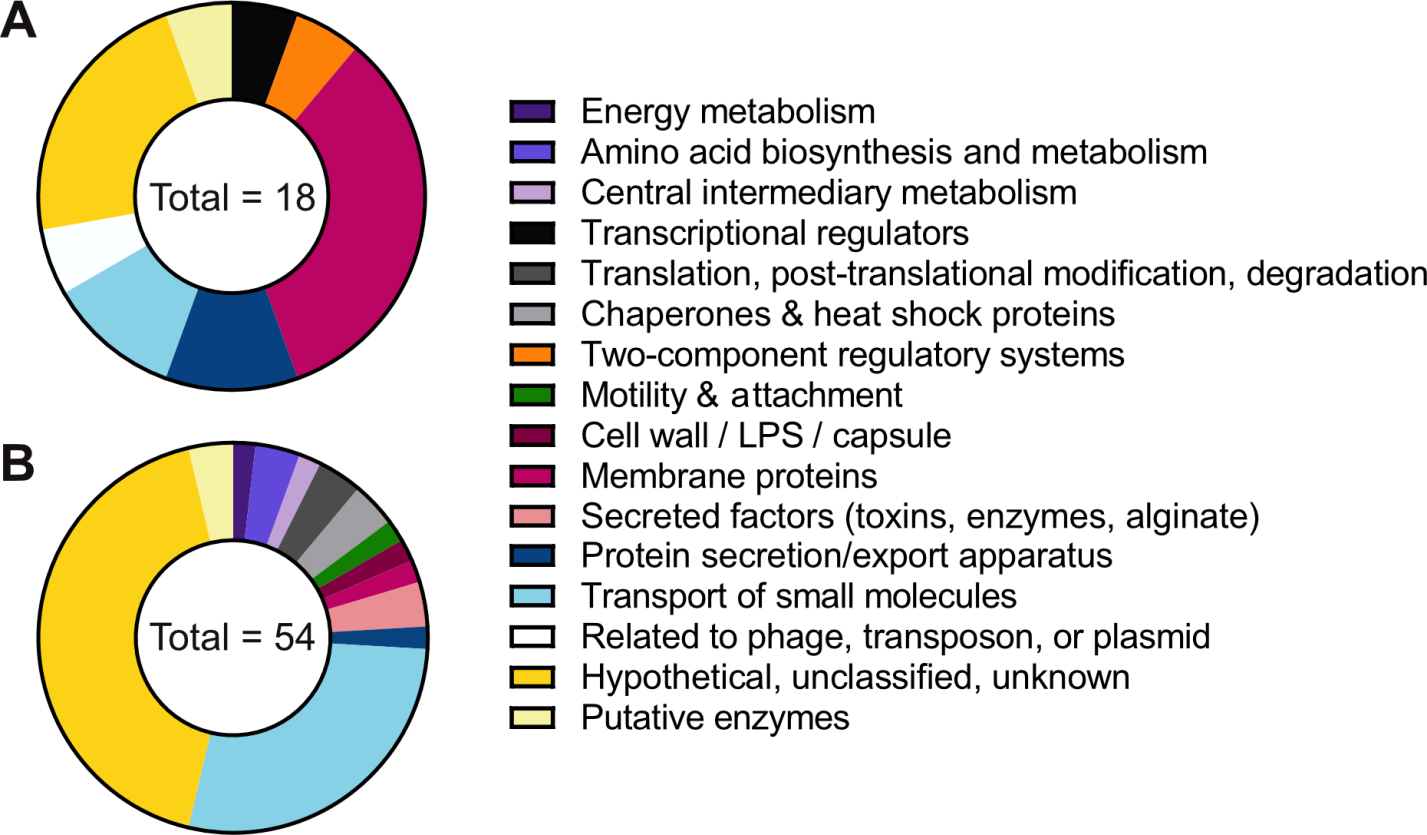
PseudoCAP functions of proteins in LESB58 with altered abundances in all β-lactam treatments. (**A**) Functions of 15 proteins that are increased ≥2-fold in abundance in all three β-lactam treatments in LESB58. (**B**) Functions of 47 proteins that are significantly decreased in abundance in all three β-lactam treatments in LESB58. The total number of annotations in each group is noted in the center of each pie chart, and these values are higher than the number of proteins, as some proteins have multiple PseudoCAP annotations. LPS, lipopolysaccharide.

Given that LESB58 has the highest MICs for the tested β-lactams, we were next interested in looking at its response to aztreonam, carbenicillin, and piperacillin in more detail, with a focus on its large response to carbenicillin. We first looked to see what changes in the proteome occurred across all three β-lactam treatments (in addition to the previously discussed increase in T6SS) and looked at the abundances of proteins known to contribute to antibiotic resistance. For LESB58 treated with aztreonam and piperacillin, only one or two proteins significantly increased in abundance (Fig. 3). We therefore examined proteins that were increased ≥2-fold in all three treatments (including proteins that did not reach the significance threshold) to identify similarities in the response to all β-lactam treatments (Table S6). Fifteen proteins were increased in abundance ≥2-fold in all three treatments, and their PseudoCAP functions are shown in Fig. 6A. We used PseudoCAP functions retrieved from the *Pseudomonas* Genome Database for this comparison because all genes/proteins in the *Pseudomonas* Genome Database have an associated PseudoCAP annotation (unlike GO and KEGG terms). These 15 proteins include ones with similar functions to those seen in the 1D annotation enrichment analysis, such as proteins involved in T6SS, membrane proteins, and transporters. In this group, we also saw two regulatory proteins, AtvR (PA2899) a transcriptional regulator, and RetS (PA4856) a two-component system sensor kinase, which were both significantly increased in abundance in the LESB58 carbenicillin treatment. To compare proteins that were decreased in abundance in all three β-lactam treatments, we looked at proteins that were significantly decreased in abundance in the Student’s *t*-tests. The PseudoCAP functions of these 47 proteins are shown in Fig. 6B. Fifteen of the proteins have the PseudoCAP annotation transport of small molecules and are known or predicted to be involved in transporting various substrates, including amino acids (seven proteins). In the 1D annotation enrichment, terms related to amino acid metabolism were enriched for proteins decreased in abundance in LESB58 treated with carbenicillin (Fig. 5). These results indicate that changes in metabolism may contribute to resistance in LESB58.

We next looked to see if and where proteins encoded in the resistome are changed in abundance on exposure to antibiotics. In our previous comparison of the proteomes of PAO1, LESlike1, and LESB58 when untreated (4-hour samples), we observed differences in the abundance of proteins with annotations related to antibiotic resistance (e.g., proteins involved in lipid A modifications and β-lactam resistance) (19). Here, we used a list of proteins retrieved from the *Pseudomonas* Genome Database under “Annotations by Category” in the list “Antimicrobial Resistance Gene Predictions,” which come from the Comprehensive Antibiotic Resistance Database (CARD) (26, 27). We also added MexX, an efflux pump component, to the list as we have previously seen that MexX is differentially abundant in the comparison of PAO1, LESlike1, and LESB58 (19). From the list of 51 proteins, 22 were significantly different in abundance in LESB58 treated with carbenicillin (Fig. S5) and only AmpC was significantly changed in abundance in more than one β-lactam treatment in LESB58 (carbenicillin and piperacillin). Most of the proteins significantly increased in abundance in LESB58 treated with carbenicillin are efflux pump components (e.g., TriABC, MexAB-OprM, MexXY, and MexVW). Only three other conditions (PAO1 with H_2_O_2_, PAO1 with TOB, and LESB58 with H_2_O_2_) had any of these proteins significantly altered in abundance compared to their untreated controls, indicating that some of these proteins may be involved in responses to different stressors. The increase in abundance of a number of resistome-encoded proteins in LESB58 treated with carbenicillin further highlights its unique response to this treatment (Fig. S5).

**Fig 7:**
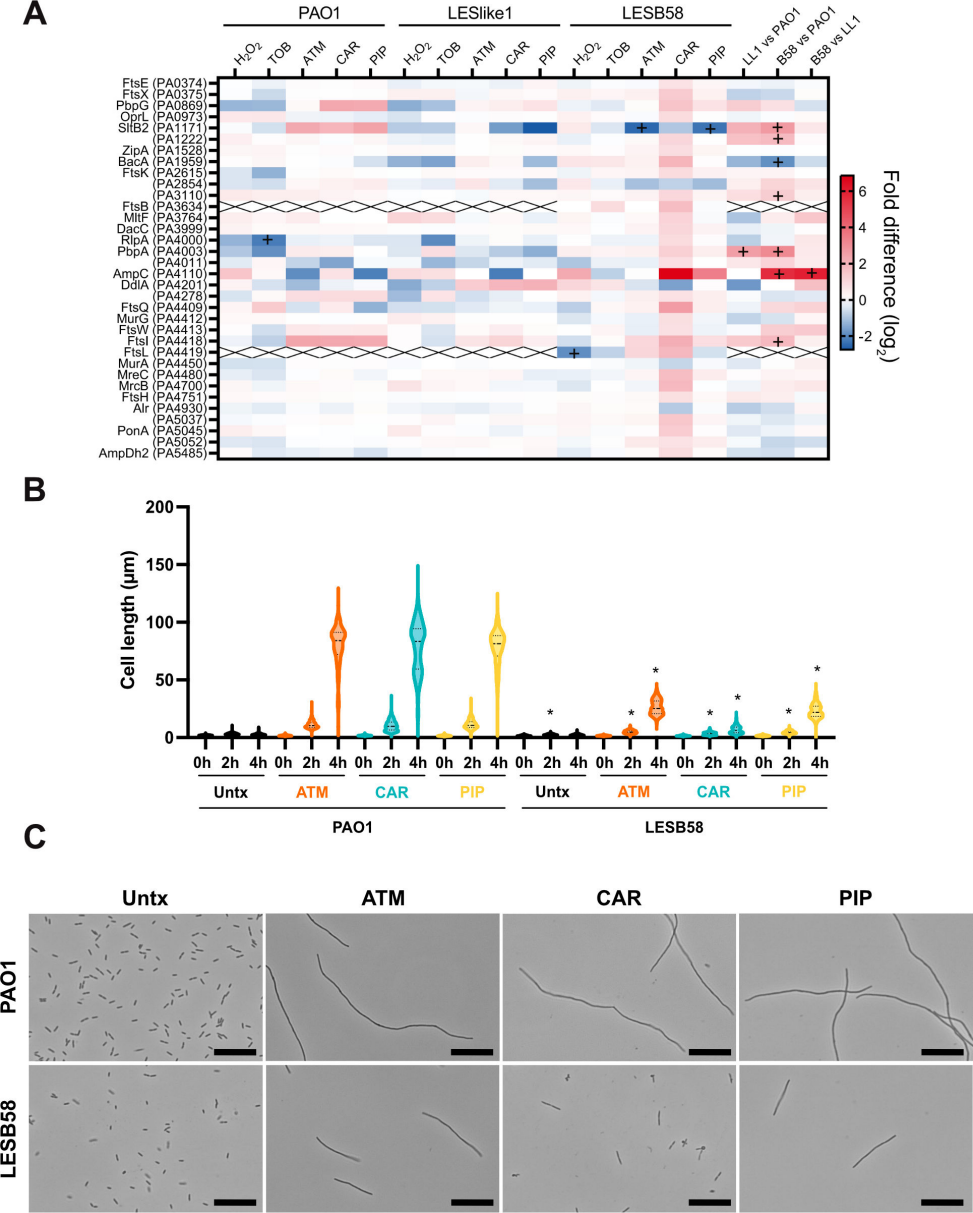
Proteins related to cell wall synthesis are increased in abundance in LESB58 treated with carbenicillin. (**A**) Heat plot indicates the fold difference in protein abundance in each comparison for proteins that were significantly different in abundance in LESB58 treated with carbenicillin. Significantly different abundances (Student’s *t*-tests, *P* ≤ 0.05, FDR = 0.05, S0 = 1) for other treatments and isolates are indicated by a plus sign (+). The last three columns present the comparisons of the 4-hour untreated controls for the three isolates (Student’s *t*-tests, *P* ≤ 0.05, FDR = 0.01, S0 = 1) previously analyzed in the reference (19). (**B**) Cell length measurements for PAO1 and LESB58 treated with β-lactams and untreated controls at 0, 2, and 4 hours. A minimum of 200 cells were measured for each sample. Solid lines represent the median cell length, and dotted lines represent the quartiles. *P < 0.05, one-way analysis of variance with Šídàk’s multiple comparisons test to compare each LESB58 sample with the corresponding PAO1 sample under the same conditions. (**C**) Representative micrographs of 4-hour samples. Bar = 20 µm. TOB, tobramycin; ATM, aztreonam; CAR, carbenicillin; PIP, piperacillin.

Since β-lactams target the cell wall, we were interested in proteins involved in cell wall synthesis and cell division, including the proteins with the various annotations enriched in LESB58 treated with carbenicillin for cell locations including “Gram-negative bacterium type cell wall.” To find all proteins related to cell wall synthesis, division, and peptidoglycan, we searched for these terms among the annotations (GO, KEGG, and UniProt keywords) for all the proteins significantly different in abundance in the LESB58 carbenicillin condition. This generated a list of 34 proteins, which were more consistently increased in abundance in LESB58 with carbenicillin compared to other treatments and isolates (Fig. 7A). This included penicillin-binding proteins and cell division proteins that may be involved in regulating septal peptidoglycan synthesis (FtsQBL) (28, 29). To see if these changes in the abundance of proteins involved in cell division and peptidoglycan synthesis were correlated with changes in cell morphology, we used phase contrast microscopy to image cells grown under the same conditions as the proteomics samples for PAO1 and LESB58 treated with aztreonam, carbenicillin, and piperacillin. Cells were imaged at 0 hour and after 2 and 4 hours of treatment. Cell length measurements (Fig. 7B) and representative micrographs of cultures after 4 hours (Fig. 7C) show that PAO1 cells are significantly more elongated under β-lactam treatments by 2 hours compared to LESB58, and this difference in cell length remained after 4 hours of treatment. In LESB58, cells became more elongated under aztreonam and piperacillin treatment but maintained a shorter length in the carbenicillin treatment.

## DISCUSSION

In this study, we used proteomics to examine both resistant and susceptible isolates of *P. aeruginosa* and their responses to antibiotic treatments. PAO1, LESlike1, and LESB58 were treated with H_2_O_2_, tobramycin, aztreonam, carbenicillin, and piperacillin. To determine the conditions for proteomics, we used time-kill assays to identify times and concentrations where the treatments affected growth, but enough cells remained for sample preparation. Other proteomics studies have used similar approaches, choosing concentrations of treatments that impact growth as measured by optical density readings. Hare et al. (14) chose concentrations of H_2_O_2_ that caused a growth perturbation, while Wüllner et al. (15) defined physiologically effective concentrations that led to 20%–50% decreases in growth within 15 minutes after treatment. In the time-kill assays we observed that the treatments affected the growth of PAO1 faster than for the LES isolates, and time points were chosen based on the PAO1 time-kills. This trend was especially true for the β-lactam treatments, where even at 1× the MIC, the CFU/mL of both LES isolates was consistent throughout the first 6 hours of the time-kill assays. It was somewhat surprising to see that LESlike1 maintained steady growth given that it is not resistant to β-lactams like LESB58. Our previous work (16) and other studies of the LES isolates (30) have shown that they grow more slowly than laboratory strains like PAO1, and it is possible that this slower growth rate may have lessened the effects of antibiotic treatment on overall cell density.

With label-free quantitative proteomics, we identified 3,019 proteins across all samples. Of these proteins, 2,631 were present in three biological replicates of at least one group of PAO1 samples, which represent ~47% of the open reading frames in PAO1 (31). The number of proteins identified is at the higher end of the range of proteome coverage reported for similar bottom-up proteomics analyses of PAO1 or clinical isolates and their responses to stress conditions [~1,100 proteins (9) to 3,967 proteins (10)]. Compared to LESlike1, PAO1 and LESB58 showed the most changes and clearer clustering of samples in the PCA. It may be tempting to attribute the lack of proteome changes in LESlike1 to treatment with lower concentrations of some antibiotics, due to its lower levels of resistance. However, the concentrations selected were relative to the resistance of each isolate, and so the concentrations used for LESlike1 were still growth inhibitory. Additionally, both PAO1 and LESlike1 were treated with much lower concentrations of β-lactams compared to LESB58, and for piperacillin they were treated with similar concentrations (8 and 4 µg/mL). LESlike1 is more susceptible to β-lactams, but it in fact has a higher MIC for tobramycin than both PAO1 and LESB58 and so was treated at a higher concentration of tobramycin. Despite this, there were no proteins with significantly different abundances in LESlike1 treated with tobramycin. One might expect that higher concentrations of antibiotics would result in more proteome changes (as seen for LESB58 and carbenicillin), but this was not always observed here. In our previous comparison of untreated PAO1, LESlike1, and LESB58, proteins involved in resistance to aminoglycosides (e.g*.,* the efflux pump MexXY) were significantly increased in abundance in the LES isolates compared with PAO1 (19). It is possible that, in the case of tobramycin, this constitutive increase in resistance to aminoglycosides resulted in few proteome changes in the LES when challenged with tobramycin. For the treatments where no proteins were significantly different in abundance, additional proteomics experiments could incorporate dosage responses and time courses such as in reference (9) to find conditions that elicit significant proteome changes. Our analysis of all proteins that were increased or decreased in abundance ≥2-fold revealed that many changes were antibiotic-specific or found in only one isolate. This highlights the variability within *P. aeruginosa* strains and isolates and their response to stress conditions. However, there were smaller groups of proteins that were shared in responses among isolates (Fig. 4). Further investigation of these proteins could help identify proteins and pathways contributing to resistance across strains/isolates. Understanding their roles could provide potential targets for novel inhibitors or antibiotics or suggest possible combination antibiotic treatments.

We next focused on the responses to non-β-lactam or β-lactam treatments. In the H_2_O_2_ treatments, we observed expected changes in the proteome profiles. Previous transcriptomic and proteomic studies have identified the same alkyl hydroperoxide reductases and catalases increased in abundance in response to H_2_O_2_ in *P. aeruginosa* (14, 20, 21). In a previous transcriptomic study of PAO1 and two LES isolates (LES400 and LES431) and their response to H_2_O_2_, an increase in phage-related gene expression was observed in LES431 (32). In LES431, an increase in the expression for the gene cluster PA0611-PA0628 was observed, which overlaps with the phage-related proteins we observed increased in abundance in PAO1 and LESlike1. Prophages are often experimentally induced by DNA-damaging agents such as mitomycin C and the production of phage-related proteins could result from DNA damage caused by oxidative stress in response to H_2_O_2_ (33). Increased expression of the phage-related genes PA0614-PA0648 has also been reported in PAO1 treated with ciprofloxacin, which targets DNA synthesis (34). In agreement with our 1D annotation enrichment analysis (Fig. 5), previous studies of the effect of H_2_O_2_ treatment on PAO1 have also reported increases in iron metabolism and siderophore biosynthesis at both the transcript and protein levels (14, 20, 21). When we examined the proteins increased in abundance ≥2-fold, the most overlap between all three isolates was seen for the H_2_O_2_ treatments. This shared response among isolates and consistency with previous studies demonstrate the value of H_2_O_2_ as a stress response control.

A previous proteomics study examined the effects of tobramycin treatment on MPAO1 at various concentrations (at or near the MIC) and times (15 minutes to 6 hours) (9). The authors found that proteins with the highest fold changes at multiple concentrations and time points were a group of heat shock proteins, proteases, and amino acid catabolic enzymes, with the heat shock protein IbpA having the highest fold changes. We checked for these 15 proteins in the data and found they were identified in all three isolates treated with tobramycin. While all the proteins were increased in abundance, only IbpA was significantly increased in abundance in PAO1 with a fold change of 3.98 (log_2_ scale), and it was among the 20 proteins with the highest fold increases in LESlike1. In the previous work, the authors created single and double knockouts of *ibpA* and several other heat shock and protease genes to test whether this affected tobramycin sensitivity. Deletion of *ibpA* alone did not affect the MIC, and other mutants only exhibited a slight change in MIC, which the authors attribute to the redundant functions of multiple heat shock proteins (9). Our data provide further evidence that these proteins may contribute to the response of PAO1 to tobramycin and that IbpA may contribute to survival in the presence of tobramycin in both laboratory and clinical strains of *P. aeruginosa*.

We included three β-lactam treatments in the study, each from a different antibiotic class. This allows proteins to be identified that are important in response to antibiotics that target the cell wall, regardless of antibiotic class/chemical structure. In a recent study, Wüllner et al. (15) challenged PAO1 with 12 antibiotics and analyzed the proteome changes using 2D gel-based proteomics. Their study included two or three antibiotics for each of the five target areas in the cell to enable the identification of marker proteins for treatments that target these cellular processes and structures, which could help identify mechanisms of action for novel antibiotics or repurposed drugs against *P. aeruginosa* (15). The same study included three β-lactam treatments (meropenem, ceftazidime, and piperacillin-tazobactam). However, proteomics did not identify any marker proteins for these treatments, which they attribute to the presence of β-lactamase activity even in untreated cultures (15). Using label-free quantitative proteomics, we identified proteins significantly increased or decreased in abundance (or with ≥2-fold changes) in all three β-lactam treatments for PAO1, LESlike1, and LESB58. These groups of proteins can be further examined as potential marker proteins for the response to cell wall–targeting antibiotics in *P. aeruginosa*. Our data also provide some evidence that markers could be strain specific. For example, the hypothetical protein PA4739 was significantly increased in abundance with high fold changes (~4- to 6-fold, log_2_ scale) in all three β-lactam treatments in PAO1. PA4739 was also recently identified as a potential biomarker of PAO1 ocular infections (35). However, in LESB58, PA4739 was significantly decreased in abundance in all three β-lactam treatments and in LESlike1 was decreased in abundance in the β-lactam treatments (fold differences from −0.36 to −2.17, log_2_ scale). This highlights the need to study clinical isolates in addition to PAO1.

The final part of our analysis focused on the response of LESB58 to β-lactam treatments. Our data identify potential markers for the response of LESB58 to cell wall–targeting antibiotics, which included hypothetical, membrane, and transport proteins increased or decreased in abundance in all three β-lactam treatments (Fig. 6). In the carbenicillin treatment, the pathways affected may indicate a shift toward a chronic/biofilm lifestyle of *P. aeruginosa*. T3SS, which is associated with acute *P. aeruginosa* infection, was decreased in abundance, while proteins associated with a biofilm phenotype were increased in abundance (alginate, T6SS, and Psl) (36). The increase in T6SS was seen in all three β-lactam treatments in LESB58 and LESlike1, and the production of T6SS proteins has previously been observed in *P. aeruginosa* PAK in response to kanamycin treatment (37). We also saw that two regulators, RetS, which is involved in the switch from T3SS to T6SS (38, 39), and AtvR, which has been shown to regulate T6SS gene expression (40), were significantly increased in abundance in LESB58 treated with carbenicillin and increased >2-fold in the aztreonam and piperacillin treatments. Another antibiotic used in treating *P. aeruginosa* lung infections, azithromycin, has been shown to affect regulatory pathways in the opposite manner, preventing biofilm formation and quorum sensing and promoting acute infection characteristics (41). Investigating how different antibiotics impact regulatory pathways in *P. aeruginosa* strains could help understand the impacts of these treatments in the clinic and possibly lead to the identification of novel methods of inhibiting *P. aeruginosa* survival. LESB58 treated with carbenicillin also showed an increase in the abundance of proteins involved in cell wall synthesis and division and had a unique cell morphology (Fig. 7C). We hypothesize that LESB58 may be able to better maintain the processes of cell wall synthesis and division under β-lactam treatment and that this may contribute to antibiotic resistance in this isolate. Cell division proteins are potential targets for antibiotic development because they are involved in an essential process (42). Additionally, inhibiting proteins involved in peptidoglycan synthesis such as lytic transglycosylases can potentiate the activity of β-lactam antibiotics (43). We believe the unique cell morphology of LESB58 warrants further investigation, and the proteomics data reported here provide a basis for studying the cell wall synthesis and division proteins important to the survival of β-lactam-resistant LES isolates.

## MATERIALS AND METHODS

### Bacterial strains and treatment conditions

Laboratory strain PAO1 (44) and two LES isolates, LESlike1 (30) and LESB58 (45), were used. Bacterial cultures were exposed to H_2_O_2_ (Fisher Scientific), tobramycin (Sigma-Aldrich), aztreonam (Sigma-Aldrich), carbenicillin (Fisher Scientific), and piperacillin (Sigma-Aldrich).

### Time-kill assays

To determine the antibiotic concentrations and length of treatment for the proteomics experiment, time-kill assays were completed for PAO1, LESlike1, and LESB58 exposed to aztreonam, carbenicillin, piperacillin, tobramycin, and H_2_O_2_. MIC values for H_2_O_2_ were first determined and were 1 mM for PAO1 and LESlike1, and 2 mM for LESB58 (Fig. S6). PAO1, LESlike1, and LESB58 were exposed to each treatment at 2×, 1×, 0.75×, and 0.5× their respective MIC values in cation-adjusted Mueller-Hinton broth (CAMHB) (BD Difco). Overnight cultures were diluted to give a starting CFU/mL of ~5 × 10^5^. Untreated controls were also inoculated, and uninoculated controls were used to check sterility. Three technical replicates were set up for each of the seven time points. At 0, 1, 2, 3, 4, 6, and 20 hours, 20 µL from each well was removed and added to 180 µL of tryptic soy broth (TSB) (BD Difco) in the top row of a 96-well plate. Samples were serially diluted down the plate to generate dilutions from 10^−1^ to 10^−8^. Dilutions were then spot plated (10 µL) on tryptic soy agar (TSA) (BD Difco). Plates were incubated overnight at 37°C and colonies were counted the next day. The CFU/mL was determined for each concentration and time point by first averaging the number of colonies in the three technical replicates. The CFU/mL at each time point for the tested concentrations was then plotted to generate time-kill curves. Three biological replicates were completed for PAO1 time-kill assays, and two biological replicates were completed for the LESlike1 and LESB58 time-kill assays.

### Growth conditions and proteomics sample preparation

PAO1, LESlike1, and LESB58 were grown in the presence of aztreonam, carbenicillin, piperacillin, tobramycin, and H_2_O_2_ at 1× their respective MICs (Table 2). Cultures were treated with the β-lactams for 4 hours, and with tobramycin and H_2_O_2_ for 2 hours. Untreated control samples were harvested at both 2 and 4 hours. Sample preparation and processing were completed as previously described (19). Briefly, cultures (starting CFU/mL 2–8 × 10^7^) were grown in 20 mL of CAMHB, harvested by centrifugation, washed twice with phosphate-buffered saline (PBS, pH 7.4), and flash frozen in liquid N_2_. Four biological replicates were prepared for each sample type. Cells were lysed by three rounds of sonication in 0.1 M Tris-HCl (pH 8.5) containing a protease inhibitor cocktail (cOmplete, Mini, EDTA-free Protease Inhibitor Cocktail tablet; Roche). SDS (2%) and dithiothreitol (0.01 M) were added to the samples before incubation at 95°C and 800 rpm for 10 minutes. Iodoacetamide (0.055 M) was added before incubation for 20 minutes at room temperature. Proteins were precipitated in ice-cold 80% acetone and later digested with LysC/Trypsin (Promega). Peptides (50 µg per sample) were purified on STAGE-tips containing three layers of C18 (46).

**TABLE 2:** Concentrations of treatments used in proteomics sample preparation

Isolate	H_2_O_2_ (mM)	TOB^*a*^ (µg/mL)	ATM^*b*^ (µg/mL)	CAR^*c*^ (µg/mL)	PIP^*d*^ (µg/mL)
PAO1	1	0.5	8	64	8
LESlike1	1	32	0.5	2	4
LESB58	2	2	512	4,096	512

^*a*^ TOB, tobramycin.

^*b*^ ATM, aztreonam.

^*c*^ CAR, carbenicillin.

^*d*^ PIP, piperacillin.

### Mass spectrometry and protein identification

Samples were analyzed by nanoflow liquid chromatography (LC) on an Ultimate 3000 LC system (Thermo Fisher Scientific) online coupled to a Fusion Lumos Tribrid mass spectrometer (Thermo Fisher Scientific) through a nanoelectrospray flex-ion source (Thermo Fisher Scientific) exactly as described in the reference (19). Peptides were eluted over 90 minutes at a constant rate of 300 nL/minute using a linear gradient from 4% to 27% acetonitrile in 0.1% formic acid and introduced into the mass spectrometer (MS) by electrospray ionization. The Fusion Lumos was operated in data-dependent mode, switching automatically between one fill scan and subsequent MS/MS scans of the most abundant peaks with a cycle time of 3 seconds. LC-MS/MS was performed in the core mass spectrometry lab of Bioinformatics Solutions Inc.

Protein identification was completed using MaxQuant software (v1.6.7.0) as previously described (19). The *P. aeruginosa* PAO1 proteome and sequences from LESlike1 and LESB58 (456 sequences) that have no BLAST hit when searched against PAO1 were used for protein identification (47). FASTA files were retrieved from UniProt (https://www.uniprot.org/proteomes/, retrieved 13 January 2020). The following settings were used in MaxQuant: digestion was set to Trypsin/P, modifications were left as the defaults (variable modifications: methionine oxidation and protein *N*-term acetylation and fixed modification: carbamidomethylation of cysteine), label-free quantification (LFQ) was selected, the LFQ minimum ratio count was set to 1, the minimum number of peptides for identification was set to 2, and “match between runs” was selected. The mass spectrometry proteomics data have been deposited in the PRIDE partner repository (48) of the ProteomeXchange Consortium with identifier: PXD034831.

### Data processing and statistical analysis

Data processing and statistical analysis were performed using Perseus software (v.16.7.0) (49). The “proteingroups.txt” output file from MaxQuant was added to Perseus, and the LFQ intensities for each sample were used for the remaining analysis. The data were filtered to remove potential contaminants, proteins identified only by site, and reverse hits. LFQ intensities for the remaining proteins were log_2_ transformed. Samples for each of PAO1, LESlike1, and LESB58 were then separated into three matrices to be analyzed separately for all subsequent steps. Biological replicate number two for LESlike1 treated with piperacillin was excluded from the analysis because of the low coverage in the sample. Column correlations were completed for all samples in each of PAO1, LESlike1, and LESB58 using a Pearson correlation and visualized by hierarchical clustering using Euclidian distance. The proteins were filtered to exclude proteins that did not have valid values (non-null values) in a minimum of three biological replicates in at least one group of samples. Imputation was used to replace missing values from a normal distribution (width = 0.3, downshift = 1.8). The mean LFQ intensity, SD, and coefficient of variation were determined for each group of biological replicates for all proteins. Principal component analyses were completed at the experiment level to reveal the clustering of the biological replicates. Two-sided Student’s *t*-tests (*P* ≤ 0.05, permutation-based FDR = 0.05, S0 = 1) were used to identify proteins with significantly different abundances in treated samples compared with their corresponding untreated control (2- or 4-hour control) and were visualized with volcano plots. Functional analysis was completed using 1D annotation enrichments (Benjamini-Hochberg FDR = 0.05) based on the *t*-test differences for each comparison (50). The 1D annotation enrichment identified enriched GO terms (biological process, cellular component, and molecular function) and pathway terms (KEGG and PseudoCyc), which were retrieved from the *Pseudomonas* Genome Database (26). The results of the 1D annotation enrichment were visualized using heat plots made using GraphPad Prism software (v8.3).

### Microscopy and cell length measurements

To assess the effect of β-lactam treatments on cell length of PAO1 and LESB58, cultures were grown as for the proteomics sample preparation. PAO1 and LESB58 were grown for 4 hours untreated or in the presence of 1× their respective MIC values of aztreonam, carbenicillin, and piperacillin. Samples were taken at 0, 2, and 4 hours for phase contrast microscopy. Imaging was done using a Leica DM2000 LED light microscope with a Leica ICC50 W camera. MicrobeJ (51) was used to measure cell length. Three biological replicates were grown and imaged for each sample, with a minimum of 200 cells measured for each sample (minimum, 224; maximum, 1,264; median, 442; mean, 517 cells/sample).

## Data Availability

The mass spectrometry proteomics data have been deposited in the PRIDE partner repository of the ProteomeXchange Consortium with identifier: PXD034831.

## SUPPLEMENTAL MATERIAL

Supplemental Figures msystems.00491-23-s0001.docx Figures S1 to S6.

Table S1 msystems.00491-23-s0002.xlsx Summary statistics for PAO1, LESlike1, and LESB58.

Table S2 msystems.00491-23-s0003.xlsx PAO1 *t*-test results.

Table S3 msystems.00491-23-s0004.xlsx LESlike1 *t*-test results.

Table S4 msystems.00491-23-s0005.xlsx LESB58 *t*-test results.

Table S5 msystems.00491-23-s0006.xlsx Results of *t*-tests for isolate comparisons at 2 and 4 h.

Table S6 msystems.00491-23-s0007.xlsx Summary of overlap between beta-lactam treatments in LESB58.

ASM does not own the copyrights to Supplemental Material that may be linked to, or accessed through, an article. The authors have granted ASM a non-exclusive, world-wide license to publish the Supplemental Material files. Please contact the corresponding author directly for reuse.
