# Supplementary material for: Quantitative proteomics reveals unique responses to antimicrobial treatments in clinical Pseudomonas aeruginosa isolates
Source: mSystems. 2023 Aug 25;8(5):e00491-23. doi: 10.1128/msystems.00491-23 (PMC10654054; doi:10.1128/msystems.00491-23)
Supplement: Supplemental Figures — Figures S1 to S6. [file msystems.00491-23-s0001.docx]

**SUPPLEMENTAL FIGURES**


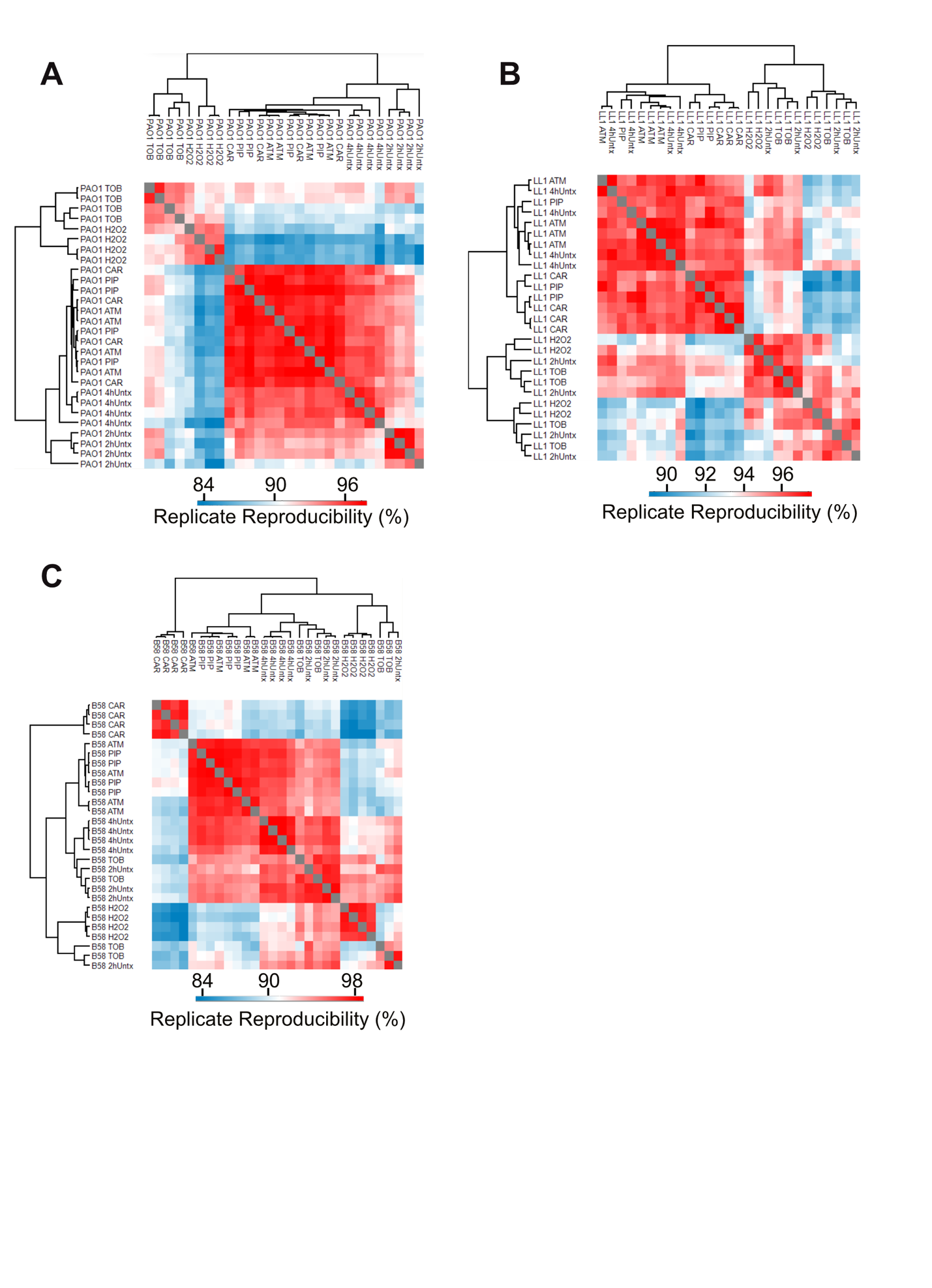


**FIG S1 Replicate reproducibility for proteomics samples.** Column correlations showing replicate reproducibility (%) for (A) PAO1, (B) LESlike1, and (C) LESB58 samples.


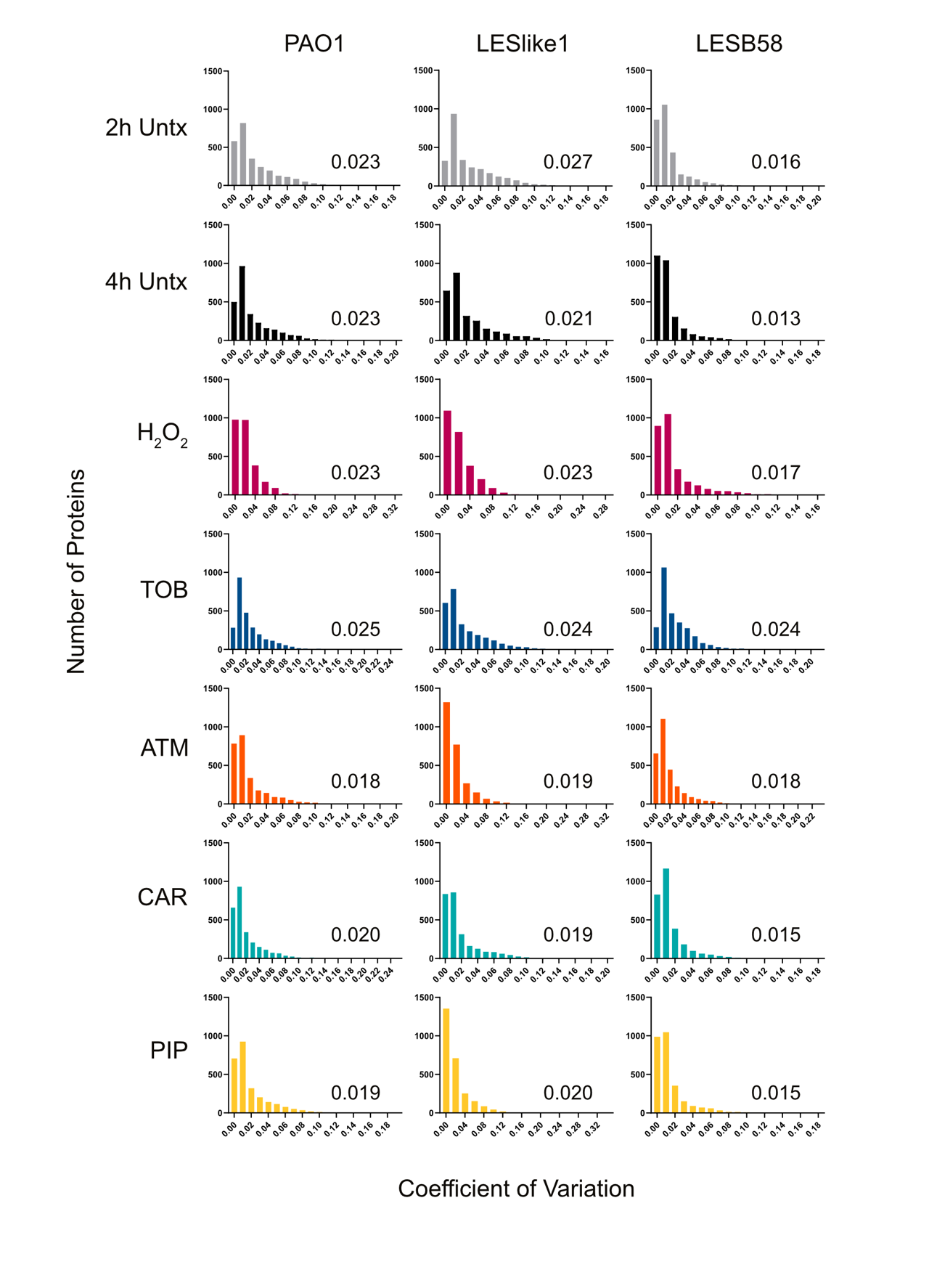


**FIG S2 Summary statistics for proteomics samples**. Frequency distributions for coefficients of variation determined for each protein in all sets of four biological replicates (PAO1, LESlike1, and LESB58 untreated controls and treated samples). The mean coefficient of variation is displayed on each graph.


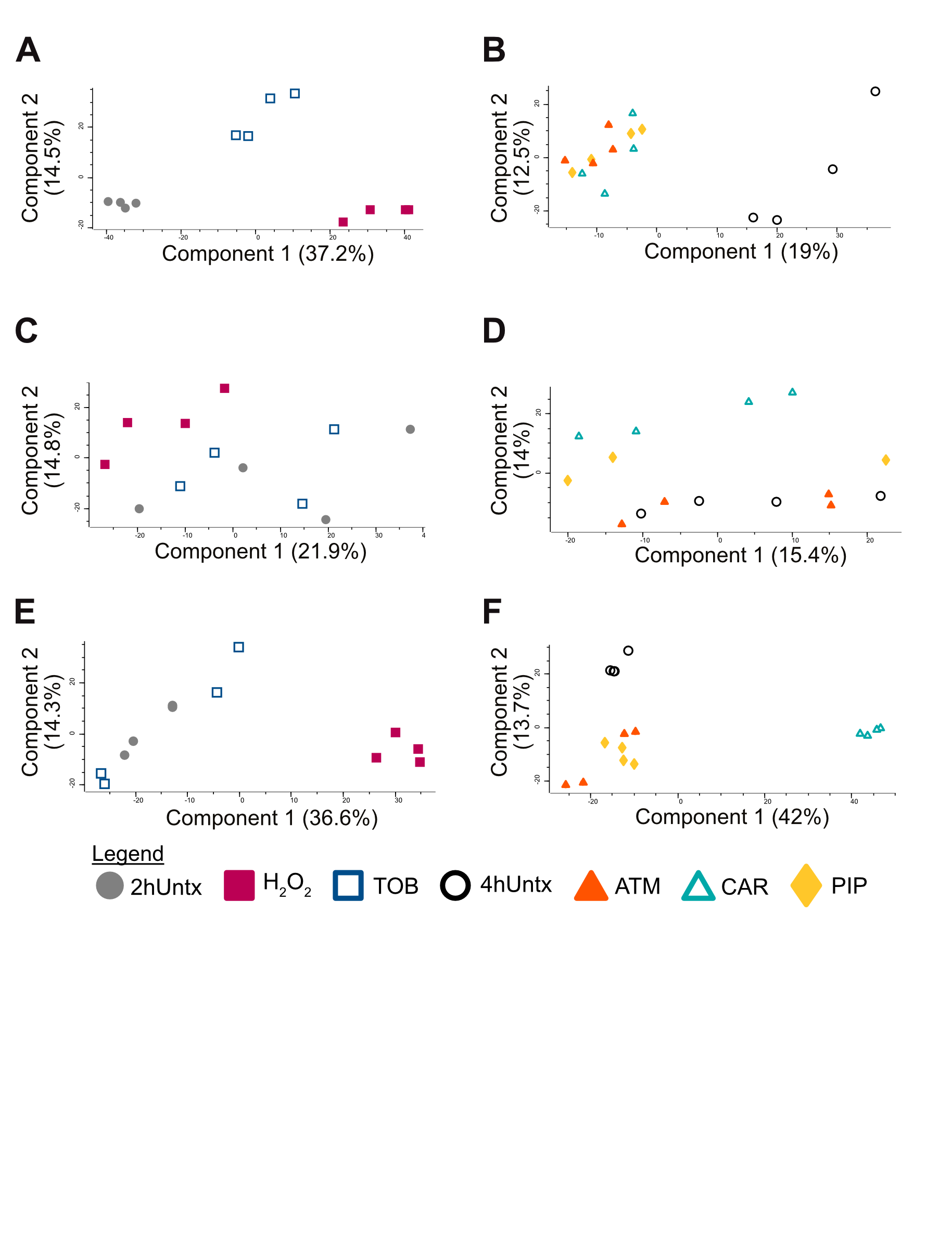


**FIG S3 Principal component analysis for 2 h and 4 h samples.** PCA at the experiment level for (A) PAO1 2 h samples, (B) PAO1 4 h samples, (C) LESlike1 2 h samples, (D) LESlike1 4 h samples, (E) LESB58 2 h samples, and (F) LESB58 4 h samples. Plots show similar clustering patterns as PCAs generated for all samples (2 h and 4 h samples) for each isolate (**Figure 2**). PCAs were generated by separately filtering for valid values and imputing missing values for 2 h or 4 h samples for each isolate. Valid value filtering removed proteins that did not have non-null values for a minimum of three biological replicates in at least one group of samples and missing values were replaced from a normal distribution (width=0.3, downshift=1.8).


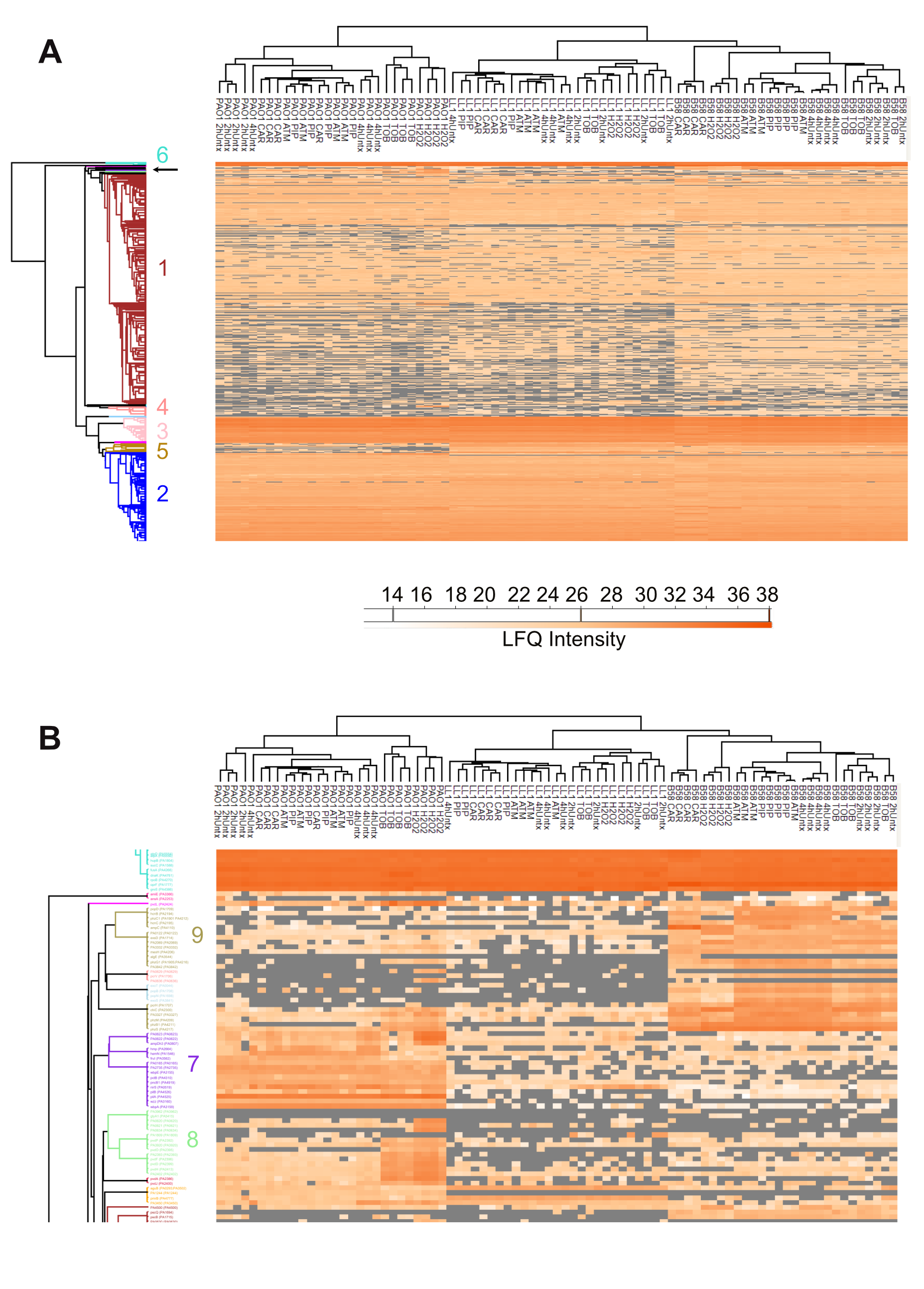


**C**

| **Cluster** | **Number of proteins** | **Trends in LFQ intensities and/or identification across samples** | **Five most abundant PseudoCAP functions in cluster**  (number of proteins with annotation) |
| --- | --- | --- | --- |
| **1** | 1813 | Proteins with moderate LFQ intensities, similar patterns of identification between isolates | Hypothetical, unclassified, unknown (693)  Transport of small molecules (171)  Putative enzymes (170)  Membrane proteins (153)  Transcriptional regulators (136) |
| **2** | 706 | Proteins identified in most samples | Hypothetical, unclassified, unknown (167)  Amino acid biosynthesis and metabolism (100)  Translation, post-translational modification, degradation (56)  Putative enzymes (55)  Energy metabolism (51) |
| **3** | 194 | Proteins with higher LFQ intensities, identified in all samples | Translation, post-translational modification, degradation (63)  Amino acid biosynthesis and metabolism (31)  Energy metabolism (26)  Carbon compound catabolism (17)  Central intermediary metabolism (15) |
| **4** | 77 | Proteins identified in few samples, more identifications in LESB58 | Hypothetical, unclassified, unknown (24)  Membrane proteins (13)  Transcriptional regulators (13)  Putative enzymes (6)  Transport of small molecules (5) |
| **5** | 73 | Proteins mainly identified in LES isolates | Transport of small molecules (16)  Hypothetical, unclassified, unknown (14)  Putative enzymes (7)  Antibiotic resistance and susceptibility (7)  Adaptation, Protection (7) |
| **6** | 28 | Proteins with high LFQ intensities, identified across all samples | Translation, post-translational modification, degradation (8)  Energy metabolism (7)  Chaperones and heat shock proteins (5)  Amino acid biosynthesis and metabolism (3)  Adaptation, Protection (3) |
| **7** | 16 | Proteins mainly identified in PAO1 | Putative enzymes (3)  Cell wall/LPS/capsule (3)  Related to phage, transposon, or plasmid (2)  Energy metabolism (2)  Biosynthesis of cofactors, prosthetic groups and carriers (2) |
| **8** | 14 | Proteins mainly identified in PAO1, higher intensities in TOB and H_2_O_2_ samples | Adaptation, Protection (5)  Related to phage, transposon, or plasmid (3)  Transport of small molecules (2)  Secreted factors (toxins, enzymes, alginate) (2)  Hypothetical, unclassified, unknown (1) |
| **9** | 13 | Proteins mainly identified in LESB58 | Adaptation, Protection (3)  Hypothetical, unclassified, unknown (3)  Protein secretion/export apparatus (2)  Central intermediary metabolism (2)  Secreted factors (toxins, enzymes, alginate) (2) |

**Figure S4 Cluster analysis of protein LFQ intensities**. (A) Heat map showing LFQ intensities for all proteins identified in a minimum of three replicates in at least one sample group (grey: protein not identified). A Pearson correlation with hierarchical clustering by Euclidian distance (number of clusters set to 20) was used to identify clusters of proteins (on the left). Nine clusters contained more than ten proteins and are labeled 1 through 9. The arrow indicates where clusters 7 to 9 are located. (B) Proteins in clusters 7, 8, and 9. (C) Summary of the number of proteins, trends in LFQ intensities/protein identification, and PseudoCAP functions for the nine highlighted clusters.


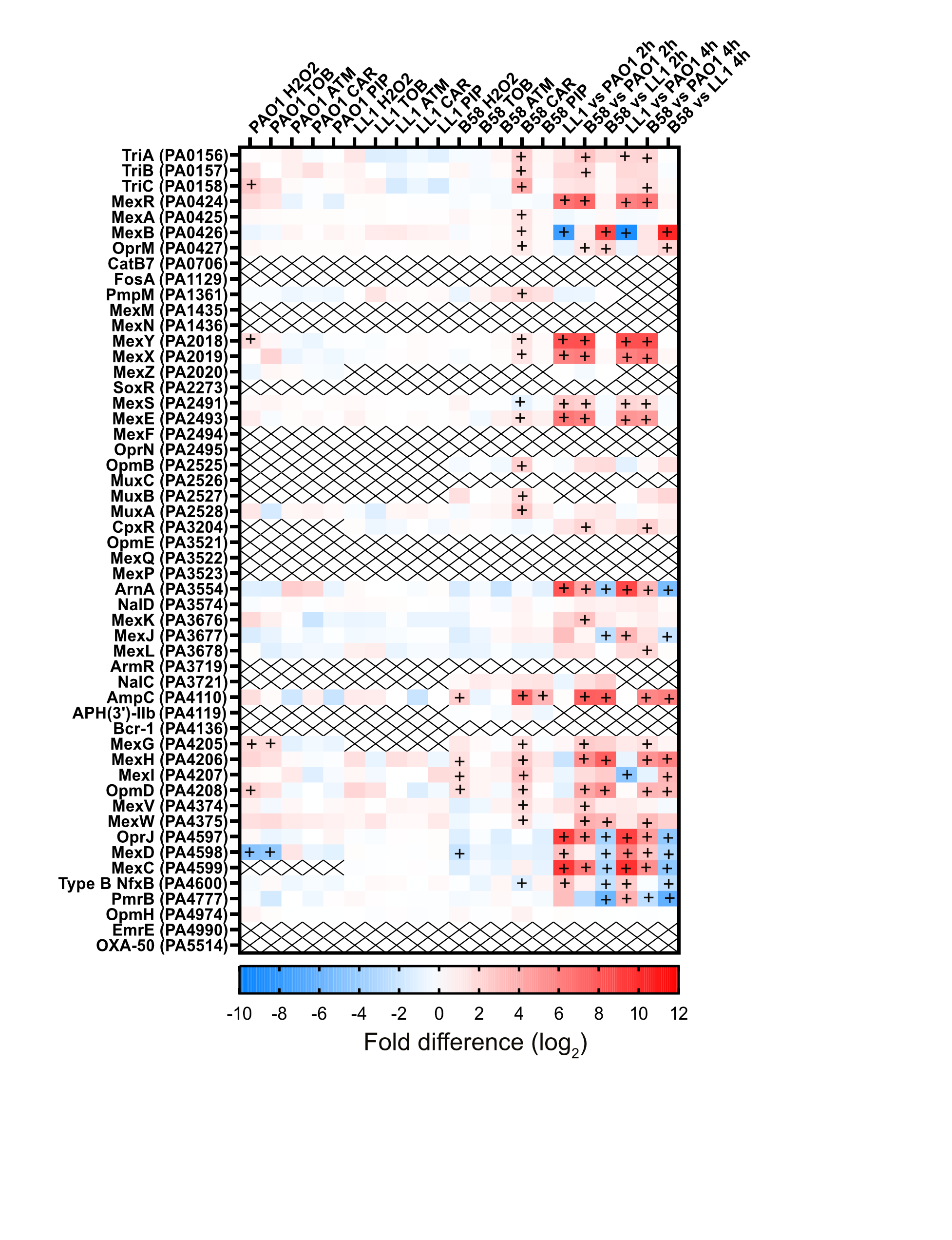


**FIG S5 CARD antimicrobial resistance gene predictions retrieved from the *Pseudomonas* Genome Database.** Heat plot indicates the fold difference in protein abundance in each comparison for proteins predicted to contribute to antimicrobial resistance (PAO1 locus number in brackets). The first fifteen columns are for the comparison of treatments with their time-matched untreated control (the focus of this study) and the final six columns are for the comparisons of the untreated samples for the three isolates at 2 h and 4 h (the 2 h and 4 h samples were analyzed separately, and the 4 h samples were previously analyzed in <https://doi.org/10.1002/prca.202100062>). Significantly different abundances (Student’s *t*-test, p≤0.05, FDR=0.05, S0=1 for the treatment comparisons, and Student’s *t*-test, p≤0.05, FDR=0.01, S0=1 for the untreated sample comparisons) are indicated by a plus sign (+). Boxes with an x indicate the protein was not identified in the group of samples where values were imputed before statistical analysis.


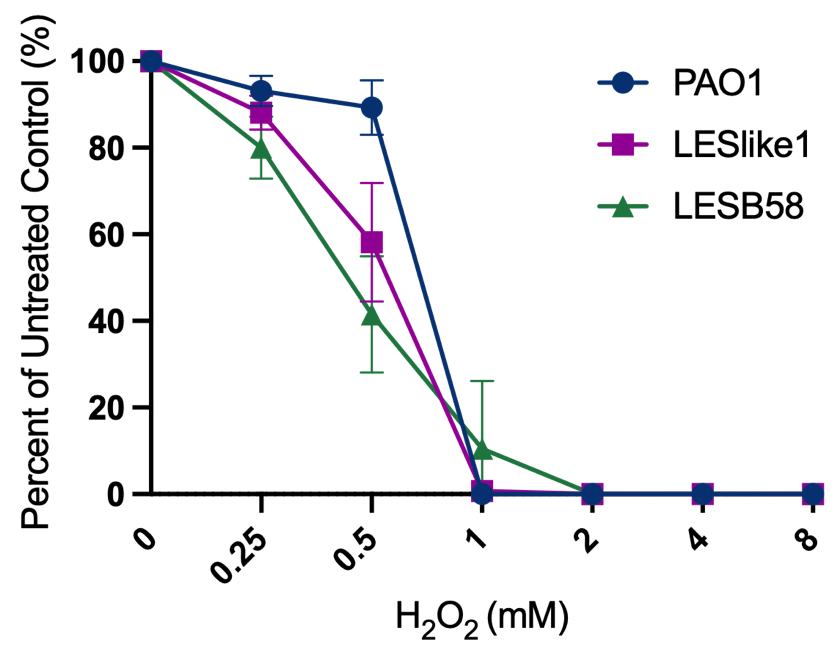


**FIG S6 H_2_O_2_ MIC assay.** The mean percent of an untreated control ± SD is shown for 3 biological replicates (three technical replicates each). MICs were determined for bacteria (2-8 × 10^5^ CFU/mL) exposed to H_2_O_2_ in CAMHB for 20 h at 37 °C. The MIC was defined as the lowest concentration of H_2_O_2_ where no visible growth was observed.
